# The potential of indigenous *Paenibacillus ehimensis* BS1 for recovering heavy crude oil by biotransformation to light fractions

**DOI:** 10.1371/journal.pone.0171432

**Published:** 2017-02-14

**Authors:** Biji Shibulal, Saif N. Al-Bahry, Yahya M. Al-Wahaibi, Abdulkadir E. Elshafie, Ali S. Al-Bemani, Sanket J. Joshi

**Affiliations:** 1 Department of Biology, College of Science, Sultan Qaboos University, Muscat, Oman; 2 Department of Petroleum and Chemical Engineering, College of Engineering, Sultan Qaboos University, Muscat, Oman; 3 Central Analytical and Applied Research Unit, College of Science, Sultan Qaboos University, Muscat, Oman; MJP Rohilkhand University, INDIA

## Abstract

Microbial Enhanced Oil Recovery (MEOR) is a potential technology for residual heavy oil recovery. Many heavy oil fields in Oman and elsewhere have difficulty in crude oil recovery because it is expensive due to its high viscosity. Indigenous microbes are capable of improving the fluidity of heavy oil, by changing its high viscosity and producing lighter oil fractions. Many spore-forming bacteria were isolated from soil samples collected from oil fields in Oman. Among the isolates, an autochthonous spore-forming bacterium was found to enhance heavy oil recovery, which was identified by 16S rDNA sequencing as *Paenibacillus ehimensis* BS1. The isolate showed maximum growth at high heavy oil concentrations within four days of incubation. Biotransformation of heavy crude oil to light aliphatic and aromatic compounds and its potential in EOR was analyzed under aerobic and anaerobic reservoir conditions. The isolates were grown aerobically in Bushnell-Haas medium with 1% (w/v) heavy crude oil. The crude oil analyzed by GC-MS showed a significant biotransformation from the ninth day of incubation under aerobic conditions. The total biotransformation of heavy crude oil was 67.1% with 45.9% in aliphatic and 85.3% in aromatic fractions. Core flooding experiments were carried out by injecting the isolates in brine supplemented with Bushnell-Haas medium into Berea sandstone cores and were incubated for twelve days under oil reservoir conditions (50°C). The extra recovered oil was analyzed by GC-MS. The residual oil recovered from core flood experiments ranged between 10–13% compared to the control experiment. The GC-MS analyses of the extra recovered oil showed 38.99% biotransformation of heavy to light oil. The results also indicated the presence of 22.9% extra aliphatic compounds in the residual crude oil recovered compared to that of a control. The most abundant compound in the extra recovered crude oil was identified as 1-bromoeicosane. The investigations showed the potential of *P*. *ehimensis* BS1 in MEOR technology by the biotransformation of heavy to lighter crude oil under aerobic and reservoir conditions. Heavy oil recovery and biotransformation to lighter components are of great economic value and a few studies have been done.

## Introduction

Crude oil is a main source of energy worldwide, which is economically important. Due to increase in energy demands and maturity of oil fields, alternative technologies should be implemented.

Heavy crude oils are called “Unconventional crude oils” because of their high viscosity and density reaching near or even greater than that of water. High concentrations of asphaltene, resins, nitrogen and sulfur containing heteroaromatics and several metals, particularly nickel and vanadium are also present in them. They are so called because they cannot be produced, transported or refined by conventional methods [1–4].

Vast deposits of heavy crude oil are found in many parts of the world. Heavy crude oil reserves are more than seven times larger than conventional oil reserves. The largest of these are in the Orinoco Oil Belt of Venezuela. Since there is depletion of conventional crude oil, in order to meet the energy demands, there is a great demand for heavy crude oil [5–7].

At present, the production and pipelining of the heavy crude oil over a significant distance is achieved by the addition of solvents, which increases the production costs [8]. This has led to the development of new methods to decrease the viscosity of heavy crude oil.

Biological processing of heavy oil is a cost effective and eco-friendly approach which provides a higher selectivity to specific reactions to upgrade heavy oil. Microbial systems which are capable of biotransforming oil fractions are used in heavy oil reservoirs for increased oil recovery by reducing the oil viscosity [9]. Many microorganisms capable of biotransforming hydrocarbons using crude oil as sole carbon source have been reported [10–15]. Several successful field trials using oil biotransforming bacteria without injection of nutrients have been reported [16, 17].

The role of spore-forming bacteria in biotransformation has already been reported, and competent *Bacillus* strains existing in many oil polluted sites have been isolated [18–22]. The spores are dormant cells which can survive in stressful environments, such as high temperature, drying and presence of acid. The dormancy period is extremely long and, so, the survival rate of the spore formers is high [23]. The biotransformation of crude oil occurs when the microbes consume heavier hydrocarbons producing lighter ones [24, 25]. The intent of this study is to demonstrate the potential of *Paenibacillus ehimensis* strain BS1 in the biotransformation of heavy crude oil (American Petroleum Institute, API = 4.57°) for MEOR using wet lab incubation method and core flood method.

*Paenibacillus ehimensis* was initially classified as *Bacillus ehimensis* based on the morphological and biochemical characteristics of the genus *Bacillus* [26]. However, it was later identified as the type species of the genus *Paenibacillus*, based on the phenotypic and chemotaxonomic characteristics, cellular lipid and fatty acid composition, 16S rDNA sequences, and phylogenetic relationship. There is no evidence in the literature to show that *P*. *ehimensis* strains indigenous to the Oman oil field have been characterized or assessed for their potential use in Enhanced Oil Recovery (EOR). In this research, the heavy crude oil biotransformation potential of *P*. *ehimensis strain* isolated from Oman oil field is reported.

## Materials and methods

All chemicals and reagents were of analytical grade and media were of microbiology grade from Sigma-Aldrich Co.

### Soil and oil sampling

Soil samples were collected randomly from oil-well contaminated sites from Southern Oman. Subsurface soil samples were collected aseptically in sterile sampling bags, properly labeled and transferred to wet lab and stored at 4°C for further studies. The samples were kindly provided by a local oil company.

### Physicochemical properties of oil

The viscosity of the heavy crude oil sample was estimated using RheolabQC Rotation Viscometer with different shear rates and the API° using DSA 5000 M density meter.

#### eTPH determination of soil samples

Extractable total petroleum hydrocarbon was estimated by adding 10g of soil sample with anhydrous sodium sulfate in a capped conical flask to remove the moisture from soil. Thirty ml of Dichloromethane (DCM) was added as extraction solvent, closed tightly and transferred to a mechanical shaker and mixed for 4–5 hours and allowed to settle for 1 hour. The solvent with the hydrocarbon was filtered through 110mm filter paper into a pre-weighted conical flask and allowed to concentrate overnight [5, 27–29]

### Culture media and cultivation

Two different bacterial enrichment media were used in the biotransformation study; Bushnell-Haas medium (BH medium) and Medium C [30, 31]. Mineral medium (MC) (pH-7±0.02) contained in (gL^-1^): NH_4_ NO_3_- 4.002; KH_2_PO_4_- 4.083; Na_2_HPO_4_- 7.119; MgSO_4_. 7H_2_O- 0.197. To this was added 1mL of trace metal solution containing (gL^-1^): CaCl_2_- 0.00077; FeSO_4_. 7H_2_O- 0.0011; MnSO_4_.4H_2_O- 0.00067; Na-EDTA- 0.00148. BH medium (pH-7±0.02) consisted of (gL^-1^): MgSO_4_- 0.2; CaCl_2_- 0.02; KH_2_PO_4_- 1.0, K_2_HPO_4_- 1.0; NH_4_ NO_3_- 1.0; FeCl_3_- 0.050. All agar plates were incubated at 40°C and the culture broths at 40°C and 160rpm in a bench- top incubator shaker.

### Isolation and maintenance of spore- forming bacterial isolates

Isolation was performed by enrichment technique [32, 33]. Soil samples (1g) collected by random sampling from each site were suspended in 10ml distilled water, vortexed thoroughly for 15 min and boiled for 30 min at 90°C to kill all the vegetative cells, and the sediments were then allowed to settle. Five ml of the supernatant served as the inoculum for the first enrichment in both media with 1% (w/v) heavy crude oil, i.e. 1g crude heavy oil in 100ml media as the solitary carbon source in 250ml conical flasks for an interval of two weeks. In a successive enrichment from these flasks, 1% v/v served as second enrichment, which was allowed to grow for a week. Negative control flasks contained no added carbon source for either of the enrichments. The dilutions from both the first and second enrichment flasks were plated on corresponding fresh agar plates uniformly spread with heavy oil for the isolation technique and all the plates were incubated at 40°C for 24h. The colonies developed on the agar plates were picked up carefully and successive streakings in fresh agar plates resulted in pure colonies. The pure cultures were preserved in 60% (v/v) glycerol stock and stored at -80°C.

### Identification of spore-forming heavy oil biotransforming *Paenibacillus ehimensis*

Among the 40 spore-forming bacteria isolated, the one which showed the maximum growth was identified as *Paenibacillus ehimensis* by molecular finger printing using Bruker’s MALDI Biotyper [34]. Identification of the strain was also done by 16S rDNA sequencing using 27F and 1492R primers of the genomic DNA, isolated using Powersoil DNA isolation kit (Mo Bio Laboratories Inc.). The amplification reaction (Polymerase Chain Reaction; PCR), was performed using T100 thermal cycler. The amplification reaction was performed on a total volume of 25μl containing: 12.5μl master mix (Promega PCR Master Mix, 2x), 9.5μl double distilled (D.D.) H2O, 1μl extracted DNA and 1μl of each primer (10pmol.). The amplification conditions were: initial denaturation step at 94°C for 3 min followed by 35 cycles of 1-min denaturation step at 94°C, 2 min annealing step at 53°C and 2 min elongation step at 72°C, and a final extension step at 72°C for 7 min. The PCR products were purified using QIAquick PCR purification kit (QIAGen). The BigDye® Terminator v3.1 Cycle Sequencing Kit (Applied Biosystems™) was used for *de novo* sequencing. The sequencing was done using 3130 XL Genetic Analyzer (Applied Biosystem, Hitachi) at Central Analytical and Applied Research Unit (CAARU), Sultan Qaboos University, and was submitted to NCBI Genbank, USA.

A dendrogram was constructed using maximum likelihood (ML) method using PHYLIP, the Phylogeny Inference Package program. The ML program uses a Hidden Markov Model (HMM) method of inferring different rates of evolution at different sites [35].

### Effect of different media and crude oil concentrations on growth of the isolate for the selection of best medium

Two different media, a mineral salt medium, Medium C [31], and Bushnell- Haas medium (BH medium) [30] with different concentrations of heavy crude oil, 1%w/v, 3% v/w, 5% v/w and 7% v/w, were used to study their effect on the growth and variation in pH under aerobic conditions. The growth of the isolate in both media was determined spectrophotometrically by reading the density at 620nm (OD_620_) every 24 hours for 10 days. The media which exhibited maximum growth of the isolate was selected for further studies. Abiotic control flasks were set up for each media. All experiments were carried out in triplicates at 40°C and 160rpm.

One-way ANOVA was conducted to determine if the heavy crude oil concentration had an effect on the growth of the isolate. Kruskal-Wallis test was used to evaluate the effect of crude oil concentration in the pH of the culture media.

### Biotransformation studies using GC-MS

*P*. *ehimensis* strain BS1 was cultured in BH medium with 1% heavy crude oil as the sole C-source for a period of 12 days to determine the biotransformation potential of the isolate under aerobic conditions. Seed culture was prepared from a single colony of the isolate, grown overnight in Luria-Bertani broth at 40°C and 160rpm, to increase the microbial density to be used in the core flood experiment. One percent of the seed culture was used as the inoculum for each of the 250ml flasks containing 100ml BH medium with 1% heavy oil. Four abiogenic control flasks were prepared with BH medium containing 1% crude heavy oil. The samples were extracted on 3^rd^, 6^th^, 9^th^ and 12^th^ days of incubation for GC-MS analysis from each of the 250ml flasks incubated at 40°C and 160rpm. All experiments were done in triplicate. The production of biosurfactant was analyzed using Drop Shape Analyzing system–DSA 100 (KRÜSS, Germany) by measuring the ST and IFT of cell-free broth. IFT was measured against Hexadecane.

#### Extraction and fractionation of residual heavy crude oil

The residual heavy oil was extracted by mixing vigorously the contents of *P*. *ehimensis* strain BS1 incubated BH medium containing 1% heavy oil with 20ml dichloromethane (DCM) in a separating funnel, allowing the mixture to separate into different fractions, and the oil fraction was carefully collected.

Different solvents, like hexane, dichloromethane, ethyl acetate and toluene, and mixtures of solvents such as Hex:DCM (4:1), Hex:DCM (1:4) and Hex:DCM (1:1) were used for the extraction of the aliphatic and aromatic compounds from the biotransformed heavy oil from the culture flask. Among the different solvents tested, Hex:DCM (1:1) proved to be the best solvent mixture and was used for rest of the study [36]. The extract was fractionated by liquid-solid chromatography using a column packed with activated silica gel G-60. The column was sequentially eluted with hexane and Hex:DCM (1:1) to yield fractions containing aliphatic and aromatic hydrocarbons, respectively.

#### GC-MS analysis

The fractions were analyzed by Waters Quattro Micro GC MS/MS with DB 5 capillary column (30m_0.32mm i.d., 0.1mm thickness) (J&W Scientific Inc., California, USA) following the EPA Method 1655 [37]. Helium was used as a carrier gas and a constant flow rate of 2ml/min was set. Injector and detector temperatures were 350°C and 370°C, respectively. Different oven temperature programs were tested, of which the following gave the best resolution: initial temperature 50°C during 1 min, raised to 350°C at the rate of 10°C/min, and held at 370°C for 1 min.

### Mineral utilization study using ICP-MS

The culture supernatant from each consecutive 3, 6, 9 and 12 days incubation with strain BS1 in BH medium was collected by centrifuging at 5000 x g and was filtered through 0.2μm pore-size sterile filters. The control supernatant was also treated the same way as the samples. All the samples were analyzed by inductively coupled plasma on an ICP-MS (Bruker Aurora M90).

### Core flooding experiment

The core flooding experiment assessed the ability of the isolate to biotransform heavy crude oil to lighter fractions under anaerobic conditions. Berea sandstone cores were used for core flooding experiment. Oil sample used in the core flooding experiment was degassed and dehydrated. The brine was purged with nitrogen. The cores were initially cleaned in methanol using soxhlet apparatus. The cleaned cores were dried at 80°C for 24 hours and saturated with filter sterilized formation water collected from the Oman heavy oil field for 12 hours in a desiccator under vacuum. The core was heated in the oven provided in the system to 50°C, mimicking the reservoir condition. The pore volume (PV) was estimated as the difference in the wet and dry weights of the core and was flooded with 4 PV of brine at 0.4cm^3^/min to ensure 100% brine saturation and to degas the core. The cores were then saturated with heavy crude oil until it reached the irreducible water saturation (S_wr_). The initial oil saturation was calculated volumetrically from the amount of injected oil and produced water. Secondary recovery of the heavy oil was done by flooding the core with brine at a rate of 0.4cm^3^/min, until no more oil was produced. The residual crude heavy oil in the core was measured from the volume of oil produced. The inoculum for the anaerobic biotransformation study was prepared using the 24 hours *P*. *ehimensis* culture in Luria Bertani broth (OD_620_ = 1.865, 1.49x10^9^ CFU/ml) with BH medium in the ratio of 1:4. 1.0 PV of the inoculum was injected into the core and shut in for 12 days at 50°C, after which the core was flooded with brine with the same flow rate for tertiary recovery, and the extra recovered oil was measured. A control experiment was performed under the same conditions, but without the injection of the isolate, *P*. *ehimensis* strain BS1. The extra recovered oil was analyzed using GC-MS. The effluent collected from the experiment was analyzed for the presence of the isolate, *P*. *ehimensis* strain BS1 using MALDI Biotyper. Scanning electron microscopy (SEM) analysis of the core specimen from the outlet, middle and inlet portions was done after fixation using glutaraldehyde and osmium, dehydration using ethanol, critical point drying and the specimens mounted on stubs were coated with gold using sputter coater.

### Statistical analysis

All data analyses were done using the statistical software MINITAB 14 with a maximal Type 1 error rate of 0.05. Kruskal-Wallis non-parametric test was used wherein the assumptions of ANOVA were not met.

## Results

### Physico-chemical properties of samples

**Fig 1:**
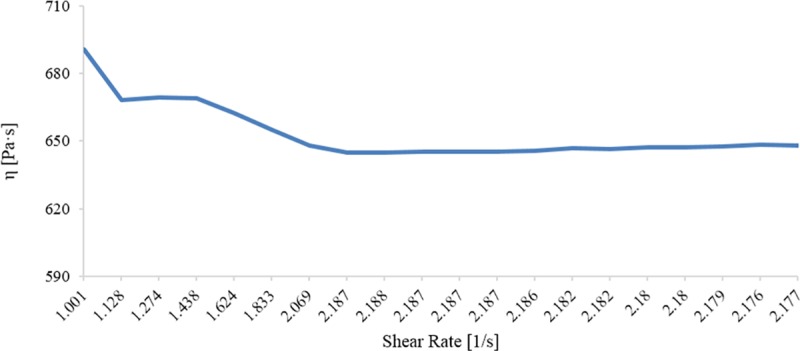
Viscosity of heavy crude oil under different shear rates.

The viscosity of the crude heavy oil was 650 *Pa*.*s* (Fig 1) and the API° was 4.57. The asphaltene content was found to be 3%. The soil sample pH was determined as 8.25 and the eTPH of the soil samples ranged from 4.2–6.2%.

### Isolation and identification of heavy crude oil utilizing spore-forming bacteria

More than forty spore-forming bacteria were isolated based on the morphology, and were screened for their ability to utilize heavy crude oil as the carbon source in two different media, BH medium and Medium C. One of the isolates which showed maximum growth on high heavy crude oil concentrations (7%w/v) was selected for the study. The isolate was identified by MALDI biotyper as *Paenibacillus* genus and by 16S rDNA sequencing; the nucleotide sequence was submitted in NCBI GenBank as *Paenibacillus ehimensis* BS1, Accession No. KP119106.1.

**Fig 2:**
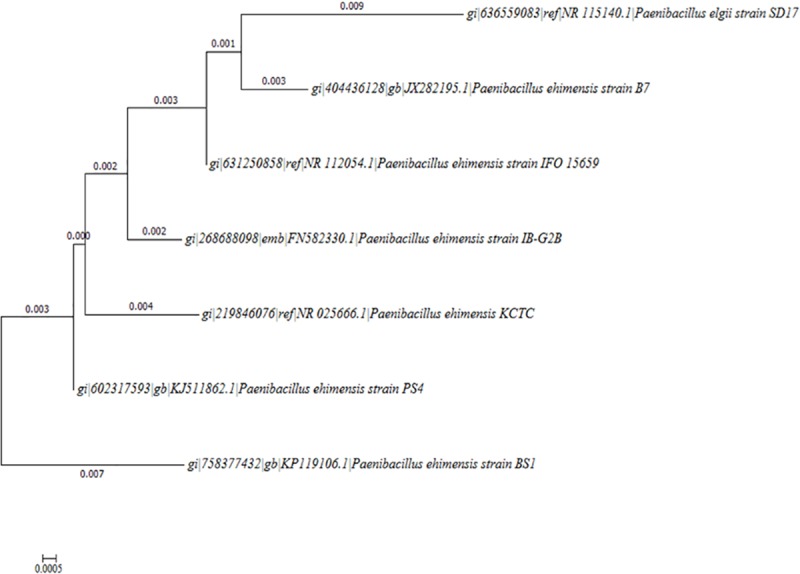
Dendrogram using maximum likelihood method. Ln Likelihood = -2263.74583. The isolate, *P*. *ehimensis* strain BS1was found to be unique from those sequences retrieved from NCBI GenBank.

The sequence was aligned with the closely related species sequences from NCBI GenBank. Bootstrap analysis was done with 1000 replications for Dendrogram (Fig 2). The branches with bootstrap values above 70% are reliable. It is evident from the dendrogram that the *P*. *ehimensis* strain BS1 from Oman is 100% different from that of others which were used to construct the phylogenetic tree reported in NCBI. The Ln Likelihood for the dendrogram was -2263.74583. The length of each tree segment in the dendrogram represents units of expected nucleotide substitutions per sites.

### Effect of different media and crude oil concentrations for the isolate growth

**Fig 3:**
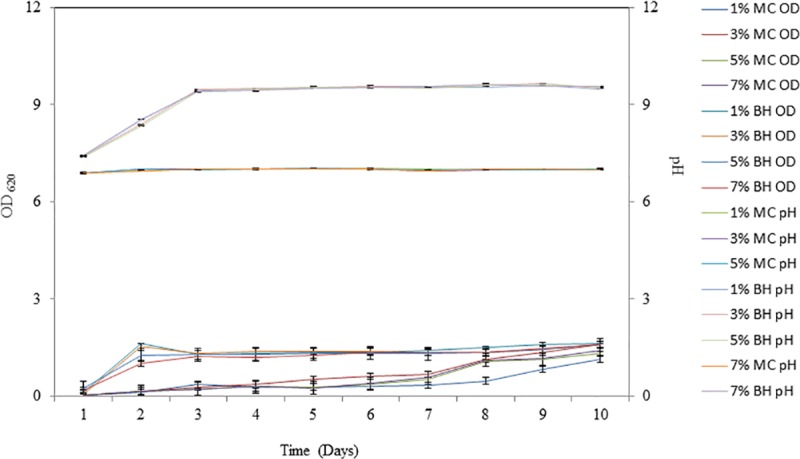
(A) Growth characteristics of *P*. *ehimensis* strain BS1 in Medium C, (B) Growth characteristics of *P*. *ehimensis* strain BS1 in BH medium. The growth of the isolate measured as OD_620_ was higher in BH medium compared to Medium C. The pH of Medium C remained the same throughout the study period, while that of the BH medium reached 9.56.

The study of growth characteristics of *P*. *ehimensis* strain BS1 in two different media, Bushnell Haas medium and Medium C, for nine days showed an increase in the growth of the isolate in BH media compared to that of the other media (Fig 3(A) and 3(B)). The pH of the Medium C remained almost the same as that of the control media (pH = 6.89), while that of the BH medium was 9.56 (Tables A and B in S1 Table).

According to ANOVA test, there was no significant effect of heavy crude oil concentration on the growth of the isolate in Medium C, F = 0.49, *p* > 0.05; R^2^ = 3.52% (Fig 3(A)) and in BH medium, F = 0.93, *p* > 0.05; R^2^ = 1.28% (Fig 3(B)). The Kruskal-Wallis test indicated that there was no significant difference in pH in Medium C with the concentration of heavy crude oil, Hc = 0.04; *p* (0.998) > 0.05 (Fig 3(A)) and no significant difference in pH in BH medium with the concentration of heavy crude oil, Hc = 0.96; *p* (0.812) > 0.05 (Fig 3(B)).

It was found that the maximum growth of *P*. *ehimensis* strain BS1 was in BH medium and this was used in the study throughout.

### Biotransformation analysis of heavy crude oil using GC-MS

**Fig 4:**
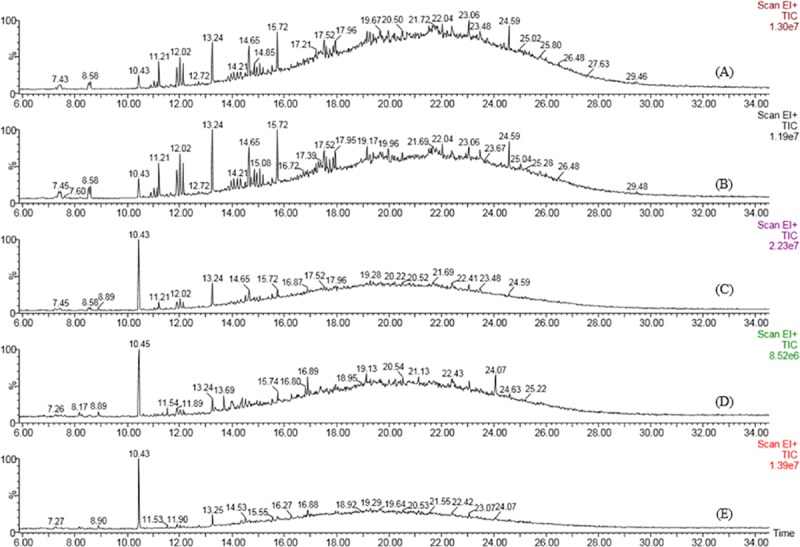
GC- MS chromatogram of heavy crude oil biotransformation by *P*. *ehimensis* strain BS1 on day 3 (B), day 6 (C), day 9 (D) and day 12 (E) compared to the control (A). The reduction in the concentrations of compounds in day 9 and day 12 in the comparative GC-MS chromatograms suggest the ability of the isolate to biofractionate crude heavy oil.

**Fig 5:**
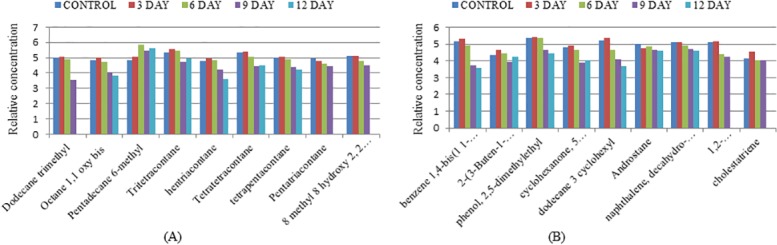
Graphical representation of fractionation of (A) aliphatic fractions (B) aromatic fractions by *P*. *ehimensis* strain BS1 on day 3, day 6, day 9 and day 12 compared to the control. The concentrations of lighter fractions are higher, compared to the heavier fractions on day 9 and 12.

The Silica G60 column purified extract from the test flasks and control flasks were analyzed using GC- MS on 3^rd^, 6^th^, 9^th^ and 12^th^ day of incubation by the strain BS1. The analysis showed 67.12% biotransformation of total crude heavy oil compared to that of abiogenic control. The results showed that the concentration of aromatic fractions on day 12 was reduced to 85.3% and that of aliphatic compounds to 45.9%, suggesting the ability of the isolate to utilize heavy fractions of hydrocarbons and convert to lighter ones. The biotransformation of heavier fractions to lighter ones accounted for an increased percentage of aliphatic compounds (Figs 4, 5A and 5B) with pentadecane (RT 10.43) as the most abundant hydrocarbon. In the control flasks, there were no differences in the concentration of compounds determined on each day (Tables A and B in S2 Table).

Analysis of variance (ANOVA) showed a significant decrease in the concentration of aromatic compounds on day9, F = 4.41, *p*(0.003) < 0.05; R^2^ = 16.39%. Post-hoc comparison using Tukey HSD showed that the concentration of compounds identified using GC-MS on day 9 and day 12 are significantly different from that of the control and also confirmed that the biotransformation rate was maximum on day 9 as the concentrations did not differ in either day 9 or day 12.

No biosurfactant production was observed in oil containing BH medium throughout the study period.

### Elemental utilization study using ICP MS

The ICP MS analysis of BH medium incubated with *P*. *ehimensis* strain BS1 showed that the most utilized element was Fe^2+^ with 98.2%, followed by Zn^2+^ and Al^3+^, being 88.5%, and the least being Mg^2+^ and B^3+^ with 47.6% and 37.2%, respectively (Table 1), compared to the control.

**Table 1:** ICP-MS analysis.

Sample	Be	B	Mg	Al	Mn	Fe	Cu	Zn	Sr
**BH medium (without oil)**	4.96	8.94	456.58	7.90	1.64	12.43	ND	5.71	ND
**BH medium (with oil)**	4.97	94.93	564.23	43.04	2.28	29.10	8.03	10.73	2.01
***P*. *ehimensis* BS1 incubated** **BH medium**	4.97	59.59	295.18	4.97	ND	0.52	5.72	1.23	ND

*concentration in ppm.

### Core flooding experiment

**Fig 6:**
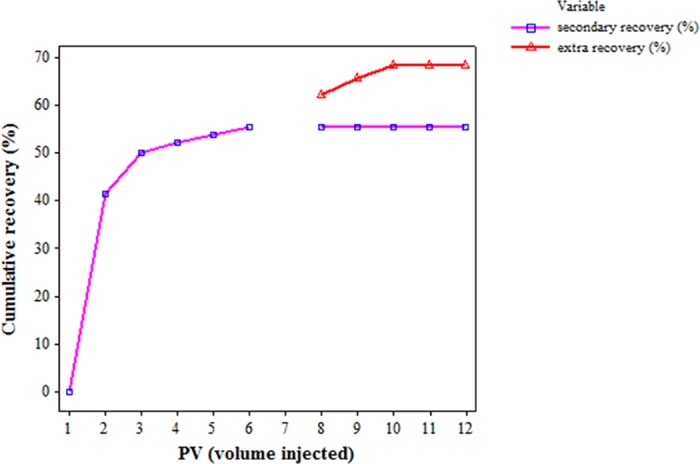
Cumulative oil recovery by *P*. *ehimensis* BS1. The extra recovery of the crude oil by the action of the isolate accounted for 10–13%.

Core flooding experiments used standard Berea sandstone cores (Table 2) to evaluate the potential of *P*. *ehimensis* strain BS1 in *in situ* MEOR. Under anaerobic conditions, the redox potential is very low and electron acceptors, such as ferric ion, nitrate and sulfate, are utilized [38, 39]. The brine contains sulfate and carbonate at various concentrations (Table 3) and, in BH medium, iron is supplemented. It was shown that the original oil in place (OIIP) in the core was 80%. The water flooding resulted in recovery of 49.2% of OIIP. The tertiary recovery by *P*. *ehimensis* strain BS1 after 12 days shut in period resulted in 10–13% extra recovery of residual oil by the biotransformation of heavy crude oil (Fig 6) compared to the control experiment, where the isolate was not injected to the core (S3 Table). This was followed by the secondary recovery of crude oil using brine, and the system was shut in for 12 days at 50°C. The control core was flooded with brine with the same flow rate as that for the test core and no extra recovery of the crude oil was observed. No pressure changes were observed during bacterial flooding. The effluent analyzed using MALDI Biotyper revealed the presence of the isolate.

**Table 2:** Basic core properties.

Core sample	Length (cm)	Diameter (cm)	Pore volume (cm^3^)	Absolute permeability (10^−2^μm^2^)	Initial water saturation (%)
1H	79.01	37.88	17.15	349.100	80.0
2H	79.08	37.88	16.58	364.505	75.8

**Table 3:** Composition of formation brine.

Density @25°C	1.05 g/cm^3^
Total salinity	9% wt.
Sodium ion	25.083 kg/m^3^
Calcium ion	3.672 kg/m^3^
Magnesium ion	0.878 kg/m^3^
Iron ion	0.045 kg/m^3^
Chloride ion	47.722 kg/m^3^
Sulfate ion	0.247 kg/m^3^
Bicarbonate ion	0.079 kg/m^3^
Filtration unit	0.45μm

**Fig 7:**
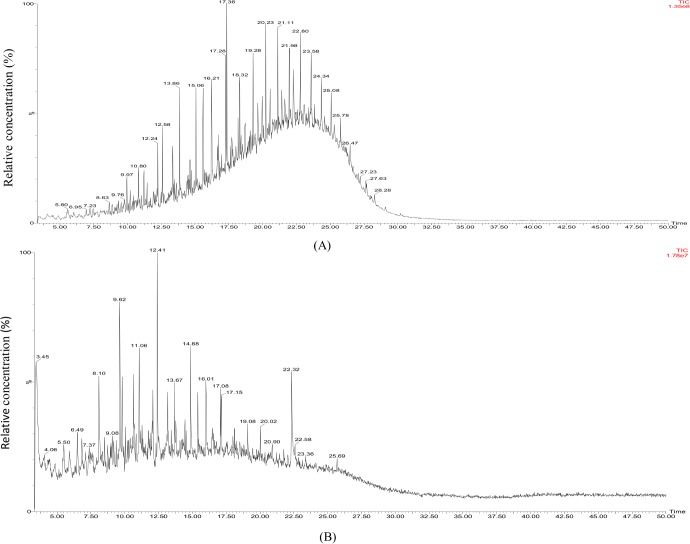
GC-MS analysis of (A) Control heavy crude oil, (B) Extra recovered oil by the action of *P*. *ehimensis* strain BS1. The relative concentration of the lighter hydrocarbons is greater in extra recovered oil compared to the control, crude heavy oil.

**Fig 8:**
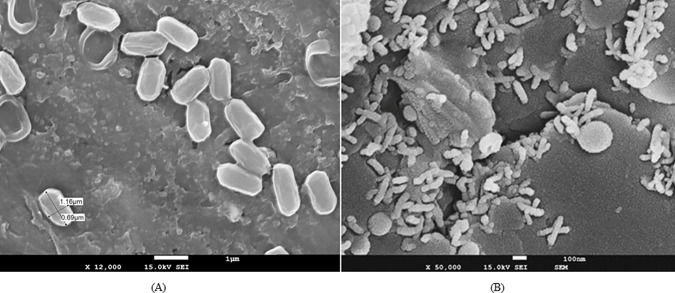
SEM of (A) *P*. *ehimensis strain BS1* in BH medium, (B) Berea core plug used in the core flood experiment. The presence of isolate colonies *P*. *ehimensis strain BS1* inside the core shows the potential of *P*. *ehimensis strain BS1* in MEOR.

The GC-MS analysis of the extra recovered residual oil showed that the biotransformation of heavy crude oil has occurred anaerobically. Most of the higher carbon fractions in the heavy oil were transformed to lighter hydrocarbons (Fig 7A and 7B) (Table 4), suggesting an increase in the flow of extra recovered oil. The compound identification of the extra recovered oil by GC-MS analysis revealed the presence of more aliphatic compounds compared to aromatic compounds (Table 4). The control for the GC-MS analysis was the abiogenic control flask having BH medium with 1% heavy crude oil, since no extra recovery was observed in control core flooding experiment. Scanning electron microscope (SEM) of core used for the anaerobic study revealed the growth of the strain BS1 under simulated reservoir conditions. Bacterial cells became slightly curved and elongated (Fig 8A and 8B).

**Table 4:** Compound identification of extra recovered oil from core flood experiment.

RT	Compound identified
3.45	3-Hexanol, 5- methyl
4.06	Octane 1,2- dibromo
5.50	Heptane 4-propyl
5.91	Octane-2,7- dimethyl
6.49	Dodecane 1,2 dibromo
7.37	Nonane-5- butyl
8.10	Heptadecadione
9.08	Heptadecane 2-methyl
9.62	11,13 dimethyl 11-tetradecen-1-ol acetate
11.06	1-Nonadecanol
12.41	1-Bromoeicosane
13.67	Eicosanol
14.88	2,15-Octadecedien-1-ol acetate
16.01	5,15- Dimethylnonadecane
17.08	Tetracosane
17.15	3-methyl tricosane
19.08	Docosane, 11- decyl
20.02	Tritriacontane
22.32	Pentatriacontane
23.36	Cyclohexane, (1-hexadecylheptadecyl)-
25.69	2,2,4,15,17,17-Hexamethyl-7,12-bis(3,5,5-trimethylhexyl)octadecane

## Discussion

The economy of some oil-producing countries depends on crude oil and a decrease in oil production will affect the countries’ development and, in turn, will increase the living costs. Increase in energy demands leads to exploration of new recovery methods such as enhanced oil recovery (EOR). Different EOR methods are currently employed, which include thermal, chemical and gas injection. Microbial enhanced oil recovery (MEOR), as suggested by Beckman in 1926 [40], is an emerging alternative for the oil recovery process.

For microbes to be used in MEOR, they should be tolerant to reservoir conditions, such as high temperature, high pressure and lack of oxygen [41]. *P*. *ehimensis* BS1 isolated from a similar environment in Oman is the first record to be reported for MEOR by biotransformation of heavy crude oil, even though the genus was reported to produce biopolymer in China [42].

In this study, heavy crude oil biotransforming spore-forming bacteria were isolated from heavy oil-contaminated soil since the ability of indigenous bacteria to mineralize crude oil hydrocarbons in oil-contaminated sites has been confirmed by many scientists [43, 44]. More than 40 spore-forming bacteria were isolated and identified by protein profiling using Bruker’s MALDI Biotyper [34]. Identification of the strain BS1 was also done by 16S rDNA sequencing and showed 99% similarity to *Paenibacillus ehimensis* in the NCBI database. Two strains showing 16S rDNA sequence homologies of 97% or higher belong to the same species [45].

The oil degradation potential was evaluated by studying the growth profile of the isolate throughout the incubation period, as increase in number of cell implies the ability of the isolate to utilize crude oil components as carbon source. This technique has been used in several studies to determine the oil degradation potential of bacteria [43, 46]. *P*. *ehimensis* strain BS1 was shown to have higher growth rate in BH medium with crude oil as carbon source compared to the C medium, the result being similar to the one already reported [47]. Crude oil can serve as a carbon source for the growth of bacteria. Higher concentrations of hydrocarbons might inhibit biodegradation by limiting nutrient or oxygen supply or by its toxic effects [48]. The growth characteristics study showed that *P*. *ehimensis* strain BS1 had significant growth in BH medium with crude heavy oil up to a concentration of 7% (w/v). The findings suggested that *P*. *ehimensis* strain BS1 is a potential candidate for crude oil biotransformation due to its relatively high tolerance to crude oil.

The rate of biotransformation of crude oil is affected by many factors, such as pH, temperature, salinity, pressure and metal ion concentration [49, 50]. It has already been reported that the fractionation of hydrocarbons is higher under slightly alkaline conditions [49, 51]. It can be inferred that the alkalinity in BH medium during the course of study increased the fractionation of heavy crude oil by *P*. *ehimensis* strain BS1.

Heavy metals greatly affect the biotransformation or biodegradation of organic pollutants by interacting with microbial enzymes or their cell walls, by interfering with the microbial general metabolism or by interrupting the functioning of the enzymes participating in the degradation of hydrocarbons [52, 53]. *P*. *ehimensis* strain BS1was found to utilize 98% of the Fe^2+^ ions present in the media containing crude heavy oil. The role of Fe^2+^ ions in microbial growth and activity is well understood. Fe^2+^ ions play an important role in microbial growth, activity and survival: they serve as a micronutrient and are used for redox-processes; they stabilize molecules through electrostatic interactions; function as components of various enzymes; and for regulation of osmotic pressure [54].

It is known that the poly aromatic hydrocarbons (PAH) present in crude oil are resistant to microbial fractionation and only a very few bacteria can act on a wide range of hydrocarbons [55]. Many strains were identified as hydrocarbon utilizers, but most of them were capable of using only a narrow range of substrates [55]. The community composition of indigenous bacteria in Gulf of Mexico beach sands indicated the abundance of members of the Gammaproteobacteria and Alphaproteobacteria as the major players in oil degradation [56]. PAH-degrading capabilities of *Arthrobacter*, *Burkholderia*, *Mycobacterium*, *Pseudomonas*, *Sphingomonas* and *Rhodococcus* were studied extensively [57]. Spore-forming consortia isolated from Oman oil fields efficiently transformed heavy crude oil into lighter hydrocarbons ranging from C11-C27 after 21 days of treatment. A mixed bacterial consortium of *Micrococcus* sp., *Bacillus* sp., *Corynebacterium* sp., *Flavobacterium* sp. and *Pseudomonas* sp. resulted in 78% degradation of crude oil after 20 days of incubation, while the maximum percentage of degradation by *Bacillus* sp. and *Micrococcus* sp. was 59% and 49%, respectively [58]. The GC-MS analysis of biotransformed heavy crude oil by *P*. *ehimensis* strain BS1 showed 67.12% biotransformation of total crude heavy oil with 85.3% reduction in aromatic fractions and the aliphatic fractions to 45.9% reduction, which suggested that *P*. *ehimensis* strain BS1 could utilize the aromatic, heavier fractions in the heavy oil and could reduce it to lighter fractions, thereby increasing the concentration of aliphatic or lower hydrocarbons. The increase in the proportion of compounds with carbon numbers between C10 to C12 and a decrease in the proportion of compounds with higher carbon compounds up to C54 were observed in the study. Similar findings were reported by other investigators with a decrease in the amount of alkanes with carbon numbers greater than 22 and an increase in the amount of alkanes between C13 and C21 in crude oil by *Pseudomonas* sp., *Actinomyces* sp. and *Bacillus* sp. [13, 24, 59]. The GC-MS analysis showed a decrease in the amount of oxygenated compounds present in the heavy crude oil by the action of the isolate, which indicated the reduction of the boiling range of the crude oil [60]. The presence of biological biomarker stearanes like androstane and cholestane were observed from GC-MS analysis, the distribution of which is of interest in oil exploration and oil forensic studies. The stearanes are believed to be derived from the cell debris of eukaryotes, mainly algae and higher plants [61].

A significant amount of heavy oil which is trapped in oil reservoirs can be recovered by biotransforming the heavier fractions to lighter ones [62]. Gudina et al. (2012) reported that *Bacillus* spp. degraded higher n-alkanes (>C27) under anaerobic conditions, which is similar to the results we obtained for *P*. *ehimensis* strain BS1. Our bacterial strain showed fractionation of higher n-alkanes having carbon numbers up to C54. *Bacillus pumilus* was isolated from long-term petroleum-contaminated soil from the Daqing oilfield which totally degraded C11-C18 hydrocarbon chains, and partially degraded C19–C24 hydrocarbons [63–65]. *Bacillus* sp. isolated from crude oil (API° 35.6)-contaminated soil samples collected from the Lingala oil field project in India was found to degrade crude oil in 60 days [66].

All these findings suggest the potential of spore-forming bacteria in enhanced oil recovery. MEOR studies with *Bacillus subtilis* showed an extra recovery of 9.6% at 37°C and 7.2% at 55°C in core flood rig studies using crude oil of 26° API, due to the combined effect of biosurfactant and its biotransforming ability [67]. An extra recovery of 16% of 13.3° API crude oil occurred after five days of incubation in core flooding experiment with *Bacillus licheniformis* [59]. *P*. *ehimensis* potential of biotransforming heavy crude oil of 4.57 API° with an extra recovery of 10–13% has not been previously reported.

The increase in the proportion of lighter hydrocarbons and decrease in the amount of higher hydrocarbon compounds in the extra recovered oil determined by GC- MS analysis in this study confirms the biotransformation ability of *P*. *ehimensis* strain BS1.

Evidence of bacterial growth inside the core was determined by SEM, which suggests that the strain BS1 was able to migrate and colonized the pores of the sandstone core and was able to biotransform the heavy crude oil. Similar results were already reported where the isolates colonized inside the cores and facilitated oil recovery [16, 68–70]. Kalish et al. reported that the Berea sandstone cores with high permeability (278–400mD) have a medium pore size distribution of 5.5–6.0μm [71]. The permeability of the Berea sandstone cores used in the study ranged between 345–365mD. The average size of the isolate, *P*. *ehimensis* BS1, as determined by SEM analysis, was 1.3μm length and 0.7μm width. The migration of the isolate was facilitated by the size dimensions of pores in the cores and the size of the isolate.

## Conclusion

*P*. *ehimensis* BS1 isolated from the Oman oil field is a promising candidate in MEOR. This is based on the ability of this strain to grow at an optimal temperature of 40–50°C (reservoir temperature). The strain BS1 can also grow in high concentration of heavy crude oil in BH medium and biotransform it to lighter ones. Fe^2+^ was the most utilized element in the medium during the course of biotransformation, the most important micronutrient needed by the isolate. Bacterial cells were able to migrate through pore spaces of sandstone rock when injected for microbial enhanced oil recovery purpose. The migration was facilitated by the size of the isolate compared to the pore size of the core. To the best of our knowledge, this is the first report of *P*. *ehimensis* having a biotransformation potential to be utilized in MEOR. Only a few studies have been done for enhanced heavy oil recovery using spore-forming bacteria.

## Supporting information

S1 Table (A) Growth characteristics of *P*. *ehimensis* strain BS1 in Medium C; (B) Growth characteristics of *P*. *ehimensis* strain BS1 in BH medium.
(DOCX)

S2 Table (A) Fractionation of aliphatic fractions by *P*. *ehimensis* strain BS1 on day 3, day 6, day 9 and day 12 compared to the control; (B) Fractionation of aromatic fractions by *P*. *ehimensis* strain BS1 on day 3, day 6, day 9 and day 12 compared to the control.
(DOCX)

S3 Table Cumulative oil recovery by *P*. *ehimensis* BS1. (DOCX)
