# Supplementary material for: The potential of indigenous Paenibacillus ehimensis BS1 for recovering heavy crude oil by biotransformation to light fractions
Source: PLoS One. 2017 Feb 14;12(2):e0171432. doi: 10.1371/journal.pone.0171432 (PMC5308839; doi:10.1371/journal.pone.0171432)
Supplement: S1 Table — (A) Growth characteristics of P. ehimensis strain BS1 in Medium C; (B) Growth characteristics of P. ehimensis strain BS1 in BH medium. (DOCX) [file pone.0171432.s001.docx]

**S1 Table A**. Growth characteristics of *P. ehimensis* strain BS1 in Medium C

|  | **pH (±0.02)** | | | | **OD620 (±0.2)** | | | |
| --- | --- | --- | --- | --- | --- | --- | --- | --- |
| **Days** | **1% crude oil** | **3% crude oil** | **5% crude oil** | **7% crude oil** | **1% crude oil** | **3% crude oil** | **5% crude oil** | **7% crude oil** |
| 0 | 6.89 | 6.89 | 6.89 | 6.89 | 0.024 | 0.024 | 0.024 | 0.024 |
| 1 | 6.96 | 7.00 | 7.00 | 6.96 | 0.120 | 0.162 | 0.141 | 0.146 |
| 2 | 7.01 | 7.01 | 6.99 | 7.00 | 0.354 | 0.271 | 0.228 | 0.228 |
| 3 | 7.02 | 7.02 | 7.01 | 7.02 | 0.263 | 0.354 | 0.314 | 0.296 |
| 4 | 7.02 | 7.03 | 7.03 | 7.02 | 0.284 | 0.512 | 0.267 | 0.256 |
| 5 | 7.03 | 7.01 | 7.01 | 7.02 | 0.313 | 0.618 | 0.365 | 0.385 |
| 6 | 7.00 | 6.96 | 6.98 | 6.96 | 0.342 | 0.678 | 0.510 | 0.577 |
| 7 | 6.98 | 6.97 | 7.00 | 7.00 | 0.474 | 1.137 | 1.076 | 1.113 |
| 8 | 6.99 | 7.01 | 6.99 | 7.01 | 0.826 | 1.352 | 1.145 | 1.153 |
| 9 | 7.00 | 6.99 | 7.01 | 6.99 | 1.145 | 1.589 | 1.326 | 1.421 |

*All values are average of three experiments

**S1 Table B**. Growth characteristics of *P. ehimensis* strain BS1 in BH medium

|  | **pH (±0.02)** | | | | **OD620 (±0.2)** | | | |
| --- | --- | --- | --- | --- | --- | --- | --- | --- |
| **Days** | **1% crude oil** | **3% crude oil** | **5% crude oil** | **7% crude oil** | **1% crude oil** | **3% crude oil** | **5% crude oil** | **7% crude oil** |
| 0 | 7.41 | 7.40 | 7.39 | 7.43 | 0.132 | 0.074 | 0.248 | 0.179 |
| 1 | 8.53 | 8.40 | 8.35 | 8.55 | 1.625 | 1.522 | 1.268 | 1.020 |
| 2 | 9.45 | 9.46 | 9.40 | 9.40 | 1.302 | 1.325 | 1.285 | 1.229 |
| 3 | 9.48 | 9.50 | 9.46 | 9.43 | 1.321 | 1.390 | 1.299 | 1.190 |
| 4 | 9.50 | 9.54 | 9.54 | 9.50 | 1.342 | 1.385 | 1.317 | 1.267 |
| 5 | 9.52 | 9.58 | 9.52 | 9.53 | 1.337 | 1.391 | 1.326 | 1.362 |
| 6 | 9.53 | 9.56 | 9.51 | 9.55 | 1.413 | 1.353 | 1.306 | 1.360 |
| 7 | 9.52 | 9.59 | 9.61 | 9.64 | 1.495 | 1.361 | 1.338 | 1.341 |
| 8 | 9.60 | 9.66 | 9.63 | 9.56 | 1.585 | 1.430 | 1.446 | 1.461 |
| 9 | 9.48 | 9.51 | 9.53 | 9.56 | 1.634 | 1.593 | 1.588 | 1.603 |

*All values are average of three experiments
