# Supplementary material for: The potential of indigenous Paenibacillus ehimensis BS1 for recovering heavy crude oil by biotransformation to light fractions
Source: PLoS One. 2017 Feb 14;12(2):e0171432. doi: 10.1371/journal.pone.0171432 (PMC5308839; doi:10.1371/journal.pone.0171432)
Supplement: S2 Table — (A) Fractionation of aliphatic fractions by P. ehimensis strain BS1 on day 3, day 6, day 9 and day 12 compared to the control; (B) Fractionation of aromatic fractions by P. ehimensis strain BS1 on day 3, day 6, day 9 and day 12 compared to the control. (DOCX) [file pone.0171432.s002.docx]

**S2 Table A**. Fractionation of aliphatic fractions by *P. ehimensis* strain BS1 on day 3, day 6, day 9 and day 12 compared to the control.

| **Compound Identified** | **Control** | **Day 3** | **Day 6** | **Day 9** | **Day 12** |
| --- | --- | --- | --- | --- | --- |
| Benzene 1,4-bis(1 1-dimethylethyl) | 150220 | 198123 | 80285 | 5568 | 3685 |
| 2-(3-Buten-1-yl)cyclohexanone | 23141 | 44140 | 27262 | 8814 | 18125 |
| Phenol, 2,5-dimethylethyl | 233521 | 267723 | 220771 | 41952 | 28576 |
| Cyclohexanone, 5 methyl 2,1 methyl ethyl | 64517 | 75450 | 42579 | 7295 | 11471 |
| Dodecane 3 cyclohexyl | 162208 | 221628 | 46084 | 12633 | 5028 |
| Androstane | 97127 | 53407 | 70283 | 42380 | 39979 |
| Naphthalene, decahydro-1,6-dimethyl-4-(1-methylethyl)- | 132130 | 133027 | 79805 | 49247 | 40765 |
| 1,2-Benzenedicarboxylic acid, mono(2-ethylhexyl) ester | 130770 | 150902 | 24577 | 17209 | 0 |
| Cholestatriene | 13726 | 34833 | 11067 | 10950 | 0 |

**S2 Table B**. Fractionation of aromatic fractions by *P. ehimensis* strain BS1 on day 3, day 6, day 9 and day 12 compared to the control.

| **Compound Identified** | **Control** | **Day 3** | **Day 6** | **Day 9** | **Day 12** |
| --- | --- | --- | --- | --- | --- |
| Dodecane trimethyl | 98694 | 122669 | 82155 | 3333 | 0 |
| Octane 1,1 oxy bis | 72384 | 101574 | 54342 | 10726 | 6402 |
| Pentadecane 6-methyl | 72645 | 122495 | 748766 | 297879 | 427349 |
| Tritetracontane | 217891 | 370947 | 271615 | 55888 | 85731 |
| hentriacontane | 58277 | 85396 | 70193 | 15667 | 4269 |
| Tetratetracontane | 216890 | 236533 | 113808 | 27143 | 30636 |
| tetrapentacontane | 98257 | 118407 | 80911 | 25521 | 16466 |
| Pentatriacontane | 86900 | 60531 | 42789 | 26856 | 0 |
| 8 methyl 8 hydroxy 2, 2 dimethyl dodeca 5,11 dien 3 one | 133230 | 134377 | 60526 | 30723 | 0 |
