# Supplementary material for: The potential of indigenous Paenibacillus ehimensis BS1 for recovering heavy crude oil by biotransformation to light fractions
Source: PLoS One. 2017 Feb 14;12(2):e0171432. doi: 10.1371/journal.pone.0171432 (PMC5308839; doi:10.1371/journal.pone.0171432)
Supplement: S3 Table — (DOCX) [file pone.0171432.s003.docx]

**S3 Table**. Cumulative oil recovery by *P. ehimensis* BS1.

| **PV** | **cumulative RF(%)** | |
| --- | --- | --- |
|  | **Control** | **Teritiary recovery** |
| 0 | 0.00 |  |
| 1 | 25.38 |  |
| 2 | 41.54 |  |
| 3 | 46.15 |  |
| 4 | 50.00 |  |
| 5 | 51.54 |  |
| 6 | 52.31 |  |
| 7 | 53.08 |  |
| 8 | 53.85 |  |
| 9 | 54.62 |  |
| 10 | 55.38 |  |
| SHUT IN PERIOD | | |
| 11 | 55.38 | 60.56 |
| 12 | 55.38 | 62.28 |
| 13 | 55.38 | 64.01 |
| 14 | 55.38 | 65.73 |
| 15 | 55.38 | 67.45 |
| 16 | 55.38 | 68.32 |
| 17 | 55.38 | 68.32 |
| 18 | 55.38 | 68.32 |
| 19 | 55.38 | 68.32 |
| 20 | 55.38 | 68.32 |
